# Crystal structures of two 1,2,3,4-tetra­hydro­naphthalenes obtained during efforts towards the total synthesis of elisabethin A

**DOI:** 10.1107/S2056989023001226

**Published:** 2023-02-17

**Authors:** Maximilian Kaiser, Matthias Weil, Peter Gärtner, Valentin Enev

**Affiliations:** a Institute of Applied Synthetic Chemistry, Research Group for Stereoselective and Sustainable Chemistry, TU Wien, Getreidemarkt 9/E163-03-03, A-1060 Vienna, Austria; b Institute for Chemical Technologies and Analytics, Division of Structural Chemistry, TU Wien, Getreidemarkt 9/E164-05-01, A-1060 Vienna, Austria; Katholieke Universiteit Leuven, Belgium

**Keywords:** crystal structure, marine diterpenoids, absolute configuration, fused ring system, disorder

## Abstract

The two substituted 1,2,3,4-tetra­hydro­naphthalenes, methyl (*R*)-3-{(1*R*,4*S*)-6-meth­oxy-4,7-dimethyl-5,8-bis­[(triiso­propyl­sil­yl)­oxy]-1,2,3,4-tetra­hydro­naph­th­al­en-1-yl}butano­ate, C_36_H_66_O_5_Si_2_, (**2**), and methyl (*E*)-3-{(1*R*,4*S*)-8-hy­droxy-6-meth­oxy-4,7-dimethyl-5-[(triiso­propyl­sil­yl)­oxy]-1,2,3,4-tetra­hydro­naphthalen-1-yl}acrylate, C_26_H_42_O_5_Si, (**8**), crystallize in the Sohncke space groups *P*2_1_2_1_2_1_ and *P*2_1_, respectively, with the absolute structure determined on the basis of anomalous dispersion effects. The configurations of the stereo centres in the 1,2,3,4-tetra­hydro­naphthalene moiety of (**2**) and (**8**) are the same, and the conformation of the non-aromatic part of the ring system is nearly identical. In the crystal of (**2**), weak non-classical C—H⋯O inter­actions consolidate the packing, whereas in (**8**), inter­molecular O—H⋯O hydrogen-bonding inter­actions of medium-to-weak strength direct the mol­ecules into *Z*-shaped strands extending parallel to [010].

## Abstract 2

The mol­ecular structures of methyl (*R*)-3-{(1*R*,4*S*)-6-meth­oxy-4,7-dimethyl-5,8-bis­[(triiso­propyl­sil­yl)­oxy]-1,2,3,4-tetra­hydro­naphthalen-1-yl}butano­ate and methyl (*E*)-3-{(1*R*,4*S*)-8-hy­droxy-6-meth­oxy-4,7-dimethyl-5-[(triiso­propyl­sil­yl)­oxy]-1,2,3,4-tetra­hydro­naphthalen-1-yl}acrylate exhibit the same configurations of the stereo centres in the 1,2,3,4-tetra­hydro­naphthalene moiety, the conformation of which is nearly identical in the two mol­ecules.

## 1. Chemical context

Elisabethin A is a marine diterpenoid that was isolated in small amounts from a Caribbean sea whip nearly 25 ago (Rodriguez *et al.*, 1998 ▸). Structure elucidation revealed a tricyclic *cis*–*trans-*fused 5,6,6 ring system with six contiguous stereo centres and a fully substituted enedione functionality. The relative configuration of elisabethin A was determined on the basis of single-crystal X-ray diffraction data (Rodriguez *et al.*, 1998 ▸), but not the absolute configuration. As a result of the scarcity of the isolated material, an extensive biological and pharmacological testing of this promising compound was not possible, making a total synthesis indispensable. A corres­ponding study was published some years later by Heckrodt & Mulzer (2003 ▸), but the allegedly successful results were questioned shortly afterwards (Zanoni & Franzini, 2004 ▸). Some years later, a second approach to the total synthesis of elisabethin A was reported (Preindl *et al.*, 2014 ▸). However, the assertions made in the previous study (Heckrodt & Mulzer, 2003 ▸) could not be proven in the subsequent study (Preindl *et al.*, 2014 ▸). As a result, the total synthesis of elisabethin A remained unsuccessful to date.

In our efforts towards the total synthesis of elisabethin A (Kaiser *et al.*, 2022 ▸), many side and inter­mediate products were obtained (Kaiser, 2022 ▸). The syntheses and crystal structures of two of them, (**2**) and (**8**), are reported in this communication. 
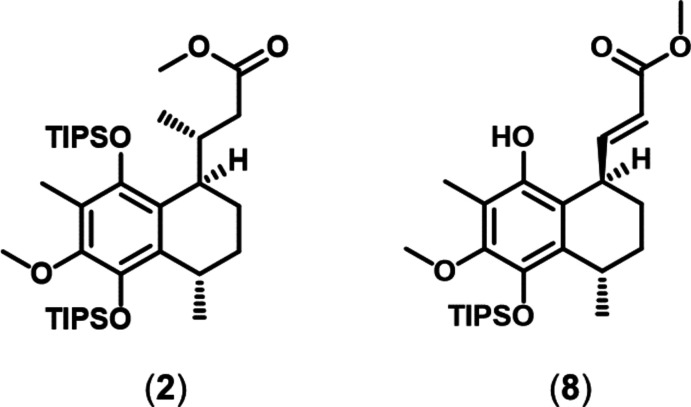


## 2. Structural commentary

**Figure 1:**
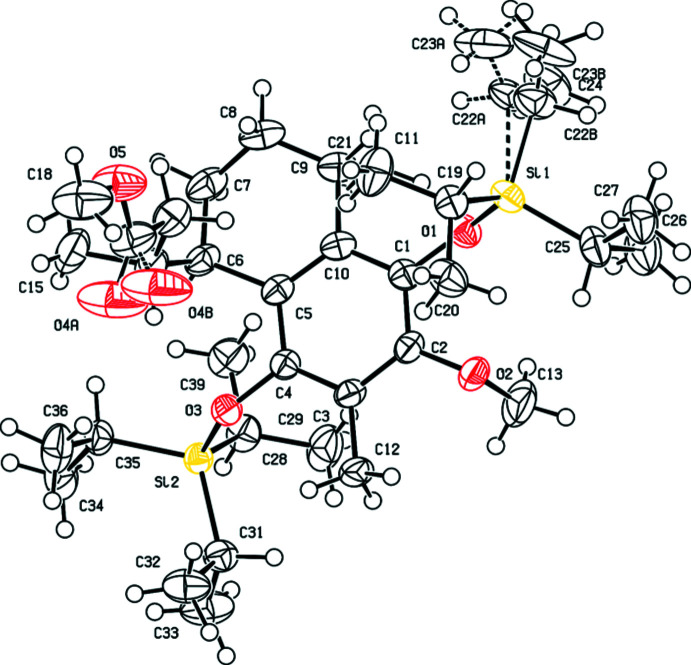
Mol­ecular structure of (**2**) with displacement ellipsoids drawn at the 50% probability level. Disorder is indicated by dashed lines (minor occupancy component).

The two compounds crystallize in Sohncke space groups, *viz. P*2_1_2_1_2_1_ for (**2**) and *P*2_1_ for (**8**). The (*R*/*S*)-configuration of the two chiral C atoms located within the 1,2,3,4-tetra­hydro­naphthalen moiety is the same in the two mol­ecules: The C6 atoms have an *R* and the C9 atoms have an *S* configuration in the two mol­ecules (Figs. 1 ▸ and 2 ▸). In (**2**), an additional chiral C atom is present, C14, which exhibits an *R* configuration. The differences between the two mol­ecules pertain to the side arms attached to C6, *viz*. butano­ate in (**2**) and acrylate in (**8**), as well as the protection of the OH group in (**8**) with a triiso­propyl­silyl group in (**2**).

**Figure 3:**
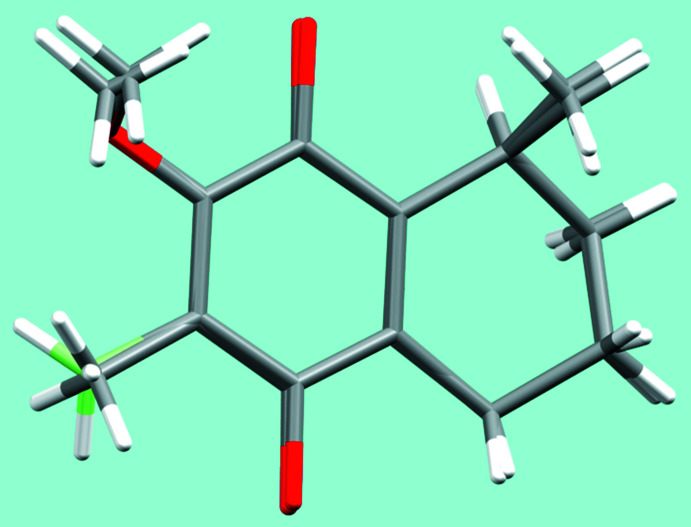
Overlay plot of the 1,2,3,4-tetra­hydro­naphthalene moiety in (**2**) and (**8**). For better distinction, the methyl C12 atom in (**2**) is given in light green.

A ring-puckering analysis (Cremer & Pople, 1975 ▸; Spek, 2020 ▸) of the non-aromatic ring part of the 1,2,3,4-tetra­hydro­naphthalen moiety revealed a half-chair conformation in both structures. The puckering parameters are similar, with individual values of *Q* = 0.444 (3) Å, *θ* = 40.2 (4)°, *φ* = 218.2 (6)° for (**2**), and *Q* = 0.5009 (13) Å, *θ* = 46.70 (14)°, *φ* = 205.60 (19)° for (**8**). In general, the two fused ring systems in (**2**) and (**8**) exhibit nearly the same conformations, as shown by the overlap of the corresponding mol­ecular entities. Only the orientation of the methyl group (C12) at the phenyl ring differs in the two mol­ecules (Fig. 3 ▸). All other bond lengths are in typical ranges, conforming with literature values (Allen *et al.*, 2006 ▸).

## 3. Supra­molecular features

**Figure 4:**
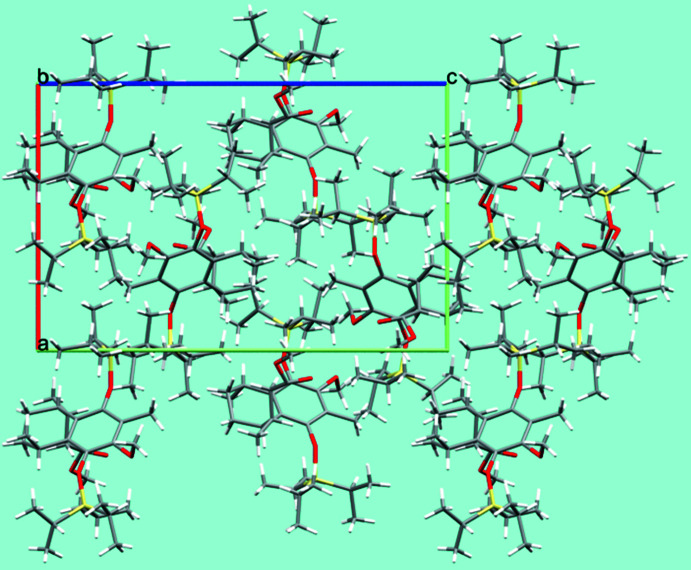
Mol­ecular packing of (**2**) in the crystal structure, shown in a view along [010]. Only the major occupancy component of the positionally disordered groups is shown; C—H⋯O hydrogen bonds are omitted for clarity.

By reason of missing polar donor groups, in (**2**) only non-classical hydrogen bonds are present, here in the form of weak C—H⋯O inter­actions (Table 1 ▸). One intra­molecular contact exists between the methine CH group (C14) of the side arm attached to C6 and an O atom, which is part of the O3—Si2 bond. An inter­molecular inter­action is developed between the methine CH group (C25) of one isopropyl chain bonded to Si2 and the carbonyl O atom (O4*B*) of the ester function attached to the side arm at C6 (Table 1 ▸). The latter hydrogen-bonding inter­action might be responsible for the positional disorder of the O4 atom. The mol­ecular packing of (**2**) is shown in Fig. 4 ▸.

**Figure 5:**
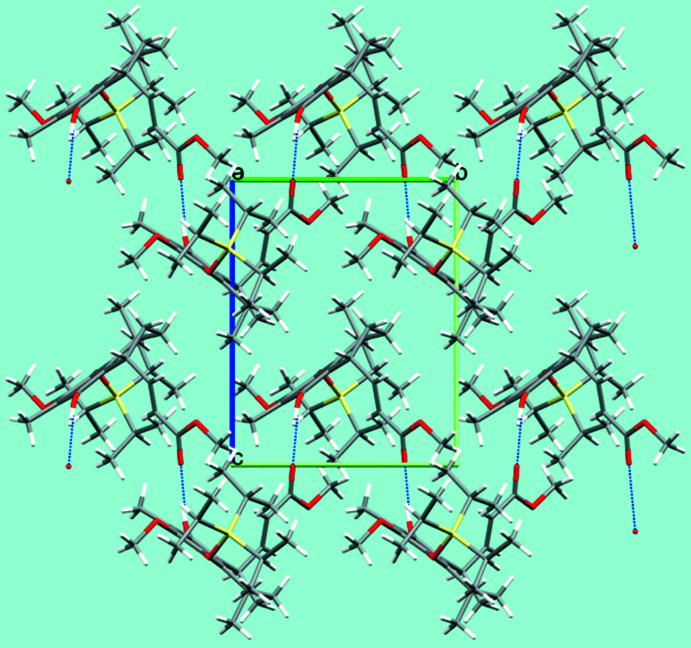
Mol­ecular packing of (**8**) in the crystal structure, shown in a view along [100]. O⋯H⋯O hydrogen bonds are shown as blue dashed lines; C—H⋯O hydrogen bonds are omitted for clarity.

In the crystal structure of (**8**), inter­molecular O—H⋯O hydrogen bonding of medium-to-weak strength is observed between the OH group (O3) and the carbonyl O atom (O4) of the ester function in the side arm attached to C6. This kind of inter­action connects neighbouring mol­ecules into *Z*-shaped strands extending parallel to [010] (Fig. 5 ▸, Table 2 ▸). Another non-classical inter­molecular C—H⋯O inter­action between a methyl H atom of the ester OCH_3_ group and the carbonyl O4 atom consolidates the packing (Table 2 ▸).

## 4. Database survey

The crystal structures of elisabethin A and D were determined by Rodriguez *et al.* (1998 ▸) and Rodriguez *et al.* (2000 ▸), respectively. A search of the Cambridge Structural Database (version 5.43, November 2022; Groom *et al.*, 2016 ▸) for related compounds on basis of the mol­ecular moiety given in Fig. 3 ▸ revealed three matches: CAXHUF (Jarvo *et al.*, 2005 ▸), CAXJER (Boezio *et al.*, 2005 ▸), and OKASUP (Ying *et al.*, 2011 ▸). In comparison with the stereo centres related to C6 and C9 in (**2**) and (**8**), CAXJER and OKASUP have the same *R* and *S* configuration, whereas CAXHUF shows an *S* and *S* configuration of the respective C atoms.

## 5. Synthesis and crystallization

**Figure 6:**
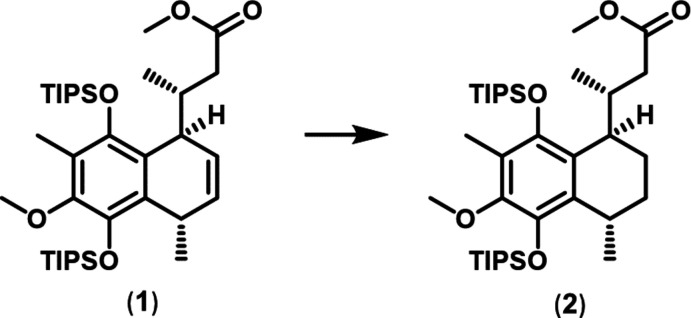
Synthesis scheme to obtain compound (**2**).

The synthesis of (**2**) is shown schematically in Fig. 6 ▸. A 50 ml Schlenk flask was equipped with 111 mg (0.175 mmol, 1 equiv.) of compound (**1**) (Kaiser *et al.*, 2022 ▸) dissolved in 15 ml of dry ethyl acetate. The colourless solution was Schlenked 10×, then Pd/C (21 mg, 19 µmol, 11 mol%) was added. The atmosphere was exchanged to H_2_ *via* vacuum/H_2_ backfill (5×) and the mixture was heated to 323 K overnight. The next day another portion of Pd/C (37 mg, 35 µmol, 20 mol%) was added and the flask was purged with fresh H_2_. Then the reaction was again heated overnight. This was repeated twice, and after 4 d, NMR quench confirmed full conversion. The atmosphere was exchanged to argon by vacuum/argon backfill (5×) and the black suspension was filtered over silica. Compound (**2**) was obtained as a pale-yellow oil that solidified on standing in 89% yield (99 mg, 0.156 mmol). Crystals of X-ray quality were obtained by slow evaporation from di­chloro­methane solution. ^1^H NMR (400 MHz, CDCl_3_): δ = 3.60 (*s*, 3H), 3.59 (*s*, 3H), 3.21–3.12 (*m*, 1H), 2.99–2.91 (*m*, 1H), 2.43–2.30 (*m*, 1H), 2.23–2.11 (*m*, 4H), 2.03 (*dd*, *J* = 14.9, 11.2 Hz, 1H), 1.96–1.85 (*m*, 1H), 1.83–1.73 (*m*, 2H), 1.49–1.41 (*m*, 1H), 1.40–1.23 (*m*, 6H), 1.14–1.01 (*m*, 39H), 0.86 (*d*, *J* = 6.8 Hz, 3H); ^13^C NMR (101 MHz, CDCl_3_): δ = 174.5, 148.0, 147.8, 141.5, 132.5, 125.2, 119.6, 60.4, 51.4, 39.6, 37.1, 34.1, 27.8, 25.8, 23.1, 18.6, 18.3, 18.2, 18.1, 18.0, 14.6, 14.1, 11.6. [α]_D_^20^ = +52.47 (*c* 1.0, CH_2_Cl_2_). Experimental ^1^H NMR and ^13^C NMR spectra are available in the electronic supporting information (ESI). Crystals of (**2**) fragmented into small parts in the cold stream of nitro­gen used for crystal cooling at temperatures < 180 K.

**Figure 7:**
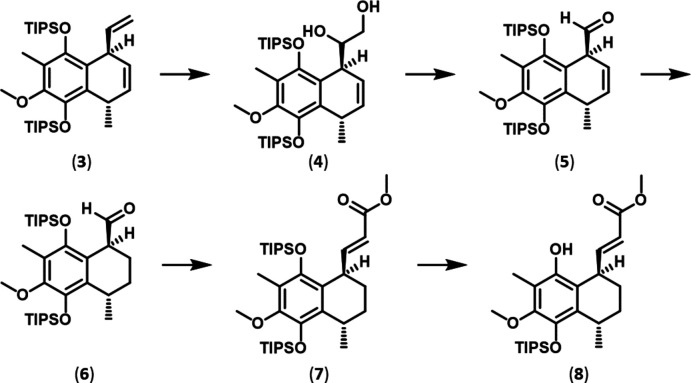
Synthesis scheme to obtain compound (**8**).

The synthetic sequence starting from (**3**) (Kaiser *et al.*, 2022 ▸) towards compound (**8**) is shown in Fig. 7 ▸. A 25 ml round-bottom flask was equipped with ester (**7**) (143 mg, 0.231 mol, 1 equiv.) and acetic acid (66 µL, 1.16 mmol, 5 equiv.), to which 0.5 ml of dry THF were added. After 5 min, TBAF (1.0 *M* in THF, 289 µl, 289 µmol, 1.25 equiv.) was added dropwise. The yellow solution was stirred at room temperature for 6 h until TLC (petroleum ether:ethyl acetate, 10:1) confirmed full conversion. The reaction was quenched with saturated NaHCO_3_ solution and the aqueous layer was extracted three times with Et_2_O. The combined organic layer was dried over MgSO_4_ and concentrated *in vacuo*. The crude material was purified by column chromatography (3.4 g silica, petroleum ether:ethyl acetate, 20:1) and (**8**) was collected as an orange oil, which solidified upon standing in 60% yield (64 mg, 0.138 mmol). Colourless crystals of X-ray quality were obtained by slow evaporation of a di­chloro­methane solution. ^1^H NMR (400 MHz, CDCl_3_): δ = 7.07 (*dd*, *J* = 15.6, 6.1 Hz, 1H), 5.44 (*dd*, *J* = 15.6, 1.6 Hz, 1H), 3.78–3.73 (*m*, 1H), 3.69 (*s*, 3H), 3.66 (*s*, 3H), 3.20–3.09 (*m*, 1H), 2.16–2.05 (*m*, 4H), 1.82–1.69 (*m*, 2H), 1.50 (*ddd*, *J* = 15.2, 5.5, 3.3 Hz, 1H), 1.39–1.29 (*m*, 3H), 1.19 (*d*, *J* = 6.9 Hz, 3H), 1.08 (*dd*, *J* = 8.8, 7.5 Hz, 18H); ^13^C NMR (101 MHz, CDCl_3_): δ = 167.4, 152.3, 148.7, 146.0, 141.4, 132.8, 121.3, 117.9, 115.5, 60.9, 51.6, 35.5, 27.8, 24.7, 22.2, 21.5, 18.3, 18.2, 14.0, 9.2. Synthetic details to obtain (**4**)–(**7**) as well as experimental ^1^H NMR and ^13^C NMR spectra for (**4**)–(**8**) are available in the ESI.

## 6. Refinement

Crystal data, data collection and structure refinement details are summarized in Table 3 ▸. Labelling of atoms for the 1,2,3,4-tetra­hydro­naphthalene moiety shown in Fig. 3 ▸ is the same in the two structures. In (**2**), one ethyl group (C22, C23) of one of the isopropyl chains bonded to Si1 is disordered over two sets of sites in a ratio of 0.541 (14):0.459 (14). The carbonyl O atom (O4) of the ester group is split over two sites in a 0.894 (11):0.106 (11) ratio. The corresponding O-atom sites were refined with the same anisotropic displacement parameters and soft restraints on the C=O bond length. In (**8**), the hydrogen atom (H1), which is part of the OH group, was located from a difference-Fourier map and was refined freely. All other H atoms in the two structures were refined using a riding model with C—H bonds fixed at calculated positions, with *U*_iso_(H) atoms set at 1.2*U*_eq_ of the parent C atom for aromatic groups and at 1.5*U*_eq_ for methyl groups.

## Supplementary Material

Crystal structure: contains datablock(s) 2, 8, global. DOI: 10.1107/S2056989023001226/vm2277sup1.cif

Structure factors: contains datablock(s) 2. DOI: 10.1107/S2056989023001226/vm22772sup2.hkl

Supporting information file. DOI: 10.1107/S2056989023001226/vm22772sup4.cml

Structure factors: contains datablock(s) 8. DOI: 10.1107/S2056989023001226/vm22778sup3.hkl

Supporting information file. DOI: 10.1107/S2056989023001226/vm22778sup5.cml

Synthetic and analytic details for compounds (2)-(8). DOI: 10.1107/S2056989023001226/vm2277sup6.docx

CCDC references: 2241092, 2241091

Additional supporting information:  crystallographic information; 3D view; checkCIF report

## Figures and Tables

**Figure 2:**
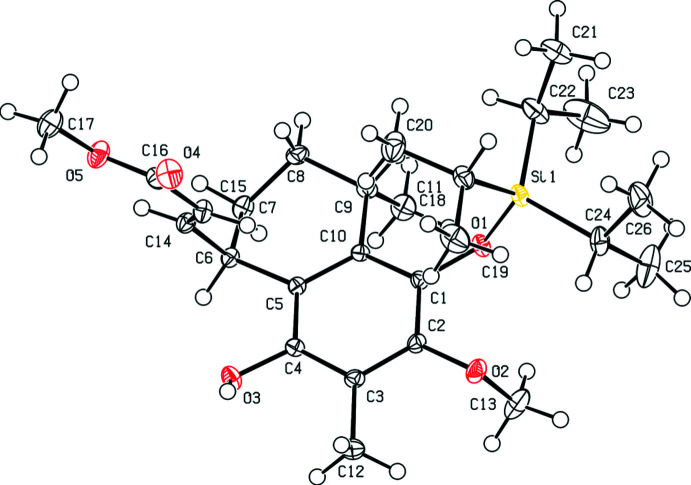
Mol­ecular structure of (**8**) with displacement ellipsoids drawn at the 50% probability level.

**Table 1:** Hydrogen-bond geometry (Å, °) for (**2**)

*D*—H⋯*A*	*D*—H	H⋯*A*	*D*⋯*A*	*D*—H⋯*A*
C14—H14⋯O3	1.00	2.46	3.115 (3)	123
C25—H25⋯O4*B* ^i^	1.00	2.46	3.312 (13)	142

Symmetry code: (i) 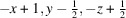 .

**Table 2:** Hydrogen-bond geometry (Å, °) for (**8**)

*D*—H⋯*A*	*D*—H	H⋯*A*	*D*⋯*A*	*D*—H⋯*A*
O3—H1⋯O4^i^	0.83 (2)	2.04 (2)	2.8658 (13)	171 (2)
C17—H17*A*⋯O4^ii^	0.98	2.57	3.471 (2)	154

Symmetry codes: (i) 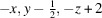 ; (ii) 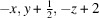 .

**Table 3:** Experimental details

	(**2**)	(**8**)
Crystal data		
Chemical formula	C_36_H_66_O_5_Si_2_	C_26_H_42_O_5_Si
*M*_r_	635.06	462.68
Crystal system, space group	Orthorhombic, *P*2_1_2_1_2_1_	Monoclinic, *P*2_1_
Temperature (K)	200	100
*a*, *b*, *c* (Å)	13.2385 (2), 14.5713 (3), 20.3505 (4)	12.0078 (7), 9.2620 (6), 12.1411 (8)
α, β, γ (°)	90, 90, 90	90, 104.0912 (14), 90
*V* (Å^3^)	3925.66 (13)	1309.66 (14)
*Z*	4	2
Radiation type	Mo *K*α	Mo *K*α
μ (mm^−1^)	0.13	0.12
Crystal size (mm)	0.4 × 0.3 × 0.2	0.6 × 0.5 × 0.4

Data collection		
Diffractometer	Bruker APEXII CCD	Bruker APEXII CCD
Absorption correction	Multi-scan (*SADABS*; Krause *et al.*, 2015 ▸)	Multi-scan (*SADABS*; Krause *et al.*, 2015 ▸)
*T*_min_, *T*_max_	0.499, 0.522	0.672, 0.747
No. of measured, independent and observed [*I* > 2σ(*I*)] reflections	27977, 9566, 7848	39668, 11727, 10749
*R*_int_	0.032	0.028
(sin θ/λ)_max_ (Å^−1^)	0.664	0.814

Refinement		
*R*[*F* ^2^ > 2σ(*F* ^2^)], *wR*(*F* ^2^), *S*	0.042, 0.112, 1.03	0.035, 0.088, 1.04
No. of reflections	9566	11727
No. of parameters	427	303
No. of restraints	20	1
H-atom treatment	H-atom parameters constrained	H atoms treated by a mixture of independent and constrained refinement
Δρ_max_, Δρ_min_ (e Å^−3^)	0.26, −0.16	0.47, −0.18
Absolute structure	Flack *x* determined using 3022 quotients [(*I* ^+^)−(*I* ^−^)]/[(*I* ^+^)+(*I* ^−^)] (Parsons *et al.*, 2013 ▸)	Flack *x* determined using 4647 quotients [(*I* ^+^)−(*I* ^−^)]/[(*I* ^+^)+(*I* ^−^)] (Parsons *et al.*, 2013 ▸)
Absolute structure parameter	−0.07 (4)	0.02 (2)

Computer programs: *APEX3* and *SAINT* (Bruker, 2018 ▸), *SHELXT* (Sheldrick, 2015*a* ▸), *SHELXL* (Sheldrick, 2015*b* ▸), *PLATON* (Spek, 2020 ▸), *Mercury* (Macrae *et al.*, 2020 ▸) and *publCIF* (Westrip, 2010 ▸).

## supplementary crystallographic information

### Methyl (R)-3-{(1R,4S)-6-methoxy-4,7-dimethyl-5,8-bis[(triisopropylsilyl)oxy]-1,2,3,4-tetrahydronaphthalen-1-yl}butanoate (2) Crystal data

**Table:**

C_36_H_66_O_5_Si_2_	*D*_x_ = 1.075 Mg m^−^^3^
*M**_r_* = 635.06	Mo *K*α radiation, λ = 0.71073 Å
Orthorhombic, *P*2_1_2_1_2_1_	Cell parameters from 9258 reflections
*a* = 13.2385 (2) Å	θ = 2.4–27.7°
*b* = 14.5713 (3) Å	µ = 0.13 mm^−^^1^
*c* = 20.3505 (4) Å	*T* = 200 K
*V* = 3925.66 (13) Å^3^	Block, light-yellow
*Z* = 4	0.4 × 0.3 × 0.2 mm
*F*(000) = 1400	

### Methyl (R)-3-{(1R,4S)-6-methoxy-4,7-dimethyl-5,8-bis[(triisopropylsilyl)oxy]-1,2,3,4-tetrahydronaphthalen-1-yl}butanoate (2) Data collection

**Table:**

Bruker APEXII CCD diffractometer	7848 reflections with *I* > 2σ(*I*)
ω–scans	*R*_int_ = 0.032
Absorption correction: multi-scan (*SADABS*; Krause *et al.*, 2015)	θ_max_ = 28.1°, θ_min_ = 2.1°
*T*_min_ = 0.499, *T*_max_ = 0.522	*h* = −17→12
27977 measured reflections	*k* = −19→19
9566 independent reflections	*l* = −26→16

### Methyl (R)-3-{(1R,4S)-6-methoxy-4,7-dimethyl-5,8-bis[(triisopropylsilyl)oxy]-1,2,3,4-tetrahydronaphthalen-1-yl}butanoate (2) Refinement

**Table:**

Refinement on *F*^2^	Hydrogen site location: inferred from neighbouring sites
Least-squares matrix: full	H-atom parameters constrained
*R*[*F*^2^ > 2σ(*F*^2^)] = 0.042	*w* = 1/[σ^2^(*F*_o_^2^) + (0.0588*P*)^2^ + 0.4115*P*] where *P* = (*F*_o_^2^ + 2*F*_c_^2^)/3
*wR*(*F*^2^) = 0.112	(Δ/σ)_max_ = 0.001
*S* = 1.03	Δρ_max_ = 0.26 e Å^−^^3^
9566 reflections	Δρ_min_ = −0.16 e Å^−^^3^
427 parameters	Absolute structure: Flack *x* determined using 3022 quotients [(*I*^+^)-(*I*^-^)]/[(*I*^+^)+(*I*^-^)] (Parsons *et al.*, 2013)
20 restraints	Absolute structure parameter: −0.07 (4)

### Methyl (R)-3-{(1R,4S)-6-methoxy-4,7-dimethyl-5,8-bis[(triisopropylsilyl)oxy]-1,2,3,4-tetrahydronaphthalen-1-yl}butanoate (2) Special details

**Table:**

Geometry. All esds (except the esd in the dihedral angle between two l.s. planes) are estimated using the full covariance matrix. The cell esds are taken into account individually in the estimation of esds in distances, angles and torsion angles; correlations between esds in cell parameters are only used when they are defined by crystal symmetry. An approximate (isotropic) treatment of cell esds is used for estimating esds involving l.s. planes.

### Methyl (R)-3-{(1R,4S)-6-methoxy-4,7-dimethyl-5,8-bis[(triisopropylsilyl)oxy]-1,2,3,4-tetrahydronaphthalen-1-yl}butanoate (2) Fractional atomic coordinates and isotropic or equivalent isotropic displacement parameters (Å^2^)

**Table:**

	*x*	*y*	*z*	*U*_iso_*/*U*_eq_	Occ. (<1)
Si1	0.41759 (5)	0.37385 (5)	0.38688 (4)	0.03659 (17)	
Si2	0.98363 (5)	0.57045 (4)	0.32279 (3)	0.03070 (15)	
O1	0.54185 (12)	0.37112 (12)	0.40106 (9)	0.0385 (4)	
O2	0.60218 (14)	0.37111 (14)	0.27186 (9)	0.0457 (5)	
O3	0.85958 (12)	0.59081 (11)	0.32245 (9)	0.0344 (4)	
O5	0.54898 (18)	0.87228 (18)	0.41882 (12)	0.0684 (7)	
O4A	0.6665 (4)	0.8485 (3)	0.34338 (19)	0.100 (2)	0.894 (11)
O4B	0.606 (3)	0.817 (3)	0.3343 (5)	0.100 (2)	0.106 (11)
C1	0.61802 (17)	0.42865 (17)	0.38090 (12)	0.0329 (5)	
C2	0.65177 (17)	0.42682 (17)	0.31672 (13)	0.0343 (5)	
C3	0.73125 (18)	0.48180 (17)	0.29553 (12)	0.0327 (5)	
C4	0.77807 (17)	0.53820 (16)	0.34189 (11)	0.0309 (5)	
C5	0.74161 (17)	0.54547 (17)	0.40669 (12)	0.0322 (5)	
C6	0.78634 (19)	0.6189 (2)	0.45193 (13)	0.0402 (6)	
H6	0.861321	0.613464	0.447376	0.048*	
C7	0.7631 (2)	0.5993 (2)	0.52442 (14)	0.0513 (8)	
H7A	0.810485	0.551779	0.540559	0.062*	
H7B	0.775231	0.655832	0.550191	0.062*	
C8	0.6557 (2)	0.5670 (2)	0.53677 (13)	0.0502 (7)	
H8A	0.608000	0.616180	0.523990	0.060*	
H8B	0.646670	0.554935	0.584280	0.060*	
C9	0.6312 (2)	0.4802 (2)	0.49818 (13)	0.0410 (6)	
H9	0.556369	0.471128	0.499058	0.049*	
C10	0.66427 (17)	0.48768 (17)	0.42665 (12)	0.0324 (5)	
C11	0.6809 (3)	0.3948 (2)	0.52833 (16)	0.0556 (8)	
H11A	0.754535	0.401154	0.526376	0.083*	
H11B	0.660249	0.340202	0.503631	0.083*	
H11C	0.659630	0.388547	0.574239	0.083*	
C12	0.7640 (2)	0.4804 (2)	0.22454 (12)	0.0439 (6)	
H12A	0.815236	0.432692	0.218248	0.066*	
H12B	0.792508	0.540288	0.212753	0.066*	
H12C	0.705552	0.467453	0.196491	0.066*	
C13	0.6480 (3)	0.2835 (2)	0.2653 (2)	0.0798 (13)	
H13A	0.715802	0.290536	0.246662	0.120*	
H13B	0.606950	0.245052	0.236215	0.120*	
H13C	0.652924	0.254385	0.308637	0.120*	
C14	0.7594 (2)	0.7160 (2)	0.42805 (15)	0.0458 (7)	
H14	0.778590	0.719060	0.380583	0.055*	
C15	0.8214 (3)	0.7899 (2)	0.46357 (19)	0.0665 (10)	
H15A	0.811237	0.849192	0.441864	0.100*	
H15B	0.893130	0.773501	0.462081	0.100*	
H15C	0.799323	0.794080	0.509441	0.100*	
C16	0.6475 (2)	0.7401 (2)	0.43193 (16)	0.0515 (7)	
H16A	0.607057	0.687497	0.415851	0.062*	
H16B	0.628812	0.751277	0.478348	0.062*	
C17	0.6229 (3)	0.8237 (2)	0.39178 (15)	0.0583 (8)	
C18	0.5192 (3)	0.9536 (3)	0.3823 (2)	0.0800 (12)	
H18A	0.577331	0.994747	0.377825	0.120*	
H18B	0.464886	0.985310	0.405891	0.120*	
H18C	0.495239	0.935582	0.338613	0.120*	
C19	0.37593 (19)	0.48929 (17)	0.35488 (13)	0.0378 (6)	
H19	0.300550	0.489674	0.358016	0.045*	
C20	0.4007 (2)	0.5064 (2)	0.28252 (15)	0.0502 (7)	
H20A	0.371280	0.565003	0.268682	0.075*	
H20B	0.372627	0.456679	0.255744	0.075*	
H20C	0.474195	0.508501	0.276759	0.075*	
C21	0.4124 (2)	0.5699 (2)	0.39687 (18)	0.0573 (8)	
H21A	0.485876	0.575789	0.392853	0.086*	
H21B	0.394539	0.559001	0.442945	0.086*	
H21C	0.380094	0.626607	0.381688	0.086*	
C22A	0.3707 (9)	0.3658 (10)	0.4775 (7)	0.044 (2)	0.459 (14)
H22A	0.431021	0.400351	0.493788	0.053*	0.459 (14)
C23A	0.3110 (11)	0.4223 (9)	0.5042 (5)	0.087 (4)	0.459 (14)
H23A	0.298531	0.473638	0.474136	0.105*	0.459 (14)
H23B	0.246953	0.391625	0.514259	0.105*	0.459 (14)
H23C	0.341121	0.445547	0.544930	0.105*	0.459 (14)
C22B	0.3470 (10)	0.3427 (10)	0.4627 (6)	0.054 (2)	0.541 (14)
H22B	0.317942	0.288003	0.439751	0.065*	0.541 (14)
C23B	0.2536 (6)	0.3706 (9)	0.4789 (5)	0.084 (4)	0.541 (14)
H23D	0.226964	0.410474	0.444167	0.101*	0.541 (14)
H23E	0.209436	0.317040	0.483896	0.101*	0.541 (14)
H23F	0.256083	0.404621	0.520375	0.101*	0.541 (14)
C24	0.4068 (3)	0.2783 (3)	0.50958 (18)	0.0696 (10)	
H24A	0.377412	0.273248	0.553643	0.104*	
H24B	0.480613	0.279209	0.512916	0.104*	
H24C	0.385674	0.225569	0.483005	0.104*	
C25	0.3855 (2)	0.28148 (18)	0.32635 (17)	0.0477 (7)	
H25	0.421102	0.298126	0.284665	0.057*	
C26	0.4238 (3)	0.1855 (2)	0.3451 (2)	0.0679 (10)	
H26A	0.384478	0.162177	0.382346	0.102*	
H26B	0.495251	0.189151	0.357480	0.102*	
H26C	0.416126	0.144034	0.307570	0.102*	
C27	0.2726 (2)	0.2770 (2)	0.3093 (2)	0.0634 (9)	
H27A	0.262105	0.232910	0.273520	0.095*	
H27B	0.249218	0.337759	0.295377	0.095*	
H27C	0.234314	0.257315	0.348031	0.095*	
C28	1.0213 (2)	0.46736 (18)	0.37291 (13)	0.0431 (6)	
H28	1.096271	0.462525	0.367691	0.052*	
C29	0.9796 (3)	0.3773 (2)	0.34638 (17)	0.0625 (9)	
H29A	1.009227	0.325901	0.370776	0.094*	
H29B	0.996667	0.371515	0.299708	0.094*	
H29C	0.906004	0.376401	0.351606	0.094*	
C39	1.0037 (3)	0.4737 (2)	0.44632 (14)	0.0531 (7)	
H39A	0.931106	0.470673	0.455364	0.080*	
H39B	1.030756	0.531983	0.462754	0.080*	
H39C	1.037963	0.422629	0.468346	0.080*	
C31	1.02275 (19)	0.54792 (19)	0.23532 (13)	0.0393 (6)	
H31	0.980197	0.495266	0.220246	0.047*	
C32	1.0000 (2)	0.6263 (2)	0.18789 (15)	0.0576 (8)	
H32A	1.047629	0.676644	0.195528	0.086*	
H32B	0.930885	0.648154	0.195089	0.086*	
H32C	1.006882	0.604369	0.142582	0.086*	
C33	1.1322 (2)	0.5166 (3)	0.22644 (17)	0.0675 (10)	
H33A	1.143151	0.498267	0.180661	0.101*	
H33B	1.145775	0.464394	0.255446	0.101*	
H33C	1.177922	0.567261	0.237511	0.101*	
C34	1.1550 (2)	0.6732 (3)	0.36574 (19)	0.0631 (9)	
H34A	1.184373	0.668205	0.321684	0.095*	
H34B	1.174878	0.619742	0.391982	0.095*	
H34C	1.179495	0.729266	0.386972	0.095*	
C35	1.03968 (19)	0.67669 (18)	0.36040 (13)	0.0382 (6)	
H35	1.013877	0.678972	0.406554	0.046*	
C36	1.0060 (3)	0.76661 (19)	0.32810 (18)	0.0617 (9)	
H36A	1.025926	0.818468	0.355877	0.092*	
H36B	0.932366	0.766383	0.322878	0.092*	
H36C	1.038005	0.772479	0.284899	0.092*	

### Methyl (R)-3-{(1R,4S)-6-methoxy-4,7-dimethyl-5,8-bis[(triisopropylsilyl)oxy]-1,2,3,4-tetrahydronaphthalen-1-yl}butanoate (2) Atomic displacement parameters (Å^2^)

**Table:**

	*U*^11^	*U*^22^	*U*^33^	*U*^12^	*U*^13^	*U*^23^
Si1	0.0305 (3)	0.0352 (3)	0.0441 (4)	0.0015 (3)	0.0059 (3)	0.0081 (3)
Si2	0.0299 (3)	0.0317 (3)	0.0306 (3)	−0.0017 (3)	−0.0030 (3)	−0.0008 (3)
O1	0.0324 (9)	0.0361 (9)	0.0470 (11)	0.0018 (8)	0.0040 (7)	0.0044 (8)
O2	0.0411 (10)	0.0489 (11)	0.0471 (11)	−0.0092 (9)	0.0042 (8)	−0.0207 (9)
O3	0.0296 (8)	0.0374 (9)	0.0361 (9)	−0.0021 (6)	0.0003 (7)	−0.0035 (7)
O5	0.0767 (15)	0.0706 (15)	0.0580 (14)	0.0324 (13)	0.0183 (11)	0.0134 (12)
O4A	0.106 (4)	0.113 (3)	0.082 (2)	0.058 (3)	0.048 (2)	0.035 (2)
O4B	0.106 (4)	0.113 (3)	0.082 (2)	0.058 (3)	0.048 (2)	0.035 (2)
C1	0.0266 (11)	0.0323 (11)	0.0397 (13)	0.0050 (9)	0.0019 (9)	0.0005 (11)
C2	0.0297 (11)	0.0350 (12)	0.0382 (13)	0.0021 (10)	−0.0021 (10)	−0.0110 (11)
C3	0.0279 (11)	0.0400 (13)	0.0302 (12)	0.0020 (10)	0.0004 (9)	−0.0083 (10)
C4	0.0266 (11)	0.0324 (11)	0.0338 (13)	0.0027 (9)	−0.0008 (9)	−0.0033 (9)
C5	0.0283 (12)	0.0369 (12)	0.0315 (12)	0.0066 (10)	−0.0035 (9)	−0.0059 (10)
C6	0.0340 (13)	0.0525 (15)	0.0342 (13)	0.0024 (12)	−0.0041 (10)	−0.0147 (12)
C7	0.0545 (18)	0.0652 (19)	0.0343 (15)	0.0059 (14)	−0.0070 (12)	−0.0147 (13)
C8	0.0549 (17)	0.0677 (19)	0.0281 (13)	0.0141 (15)	0.0041 (12)	−0.0028 (13)
C9	0.0372 (14)	0.0545 (16)	0.0312 (13)	0.0093 (12)	0.0025 (10)	0.0036 (12)
C10	0.0291 (11)	0.0383 (13)	0.0299 (12)	0.0113 (10)	−0.0007 (9)	−0.0002 (10)
C11	0.0575 (18)	0.066 (2)	0.0438 (17)	0.0130 (15)	−0.0016 (13)	0.0151 (15)
C12	0.0390 (14)	0.0621 (17)	0.0305 (13)	−0.0110 (13)	0.0019 (10)	−0.0132 (12)
C13	0.065 (2)	0.057 (2)	0.117 (3)	−0.0083 (17)	0.019 (2)	−0.048 (2)
C14	0.0499 (16)	0.0443 (15)	0.0432 (16)	−0.0013 (13)	0.0015 (12)	−0.0145 (12)
C15	0.074 (2)	0.0555 (19)	0.070 (2)	−0.0086 (17)	−0.0030 (18)	−0.0289 (18)
C16	0.0546 (18)	0.0468 (16)	0.0529 (18)	0.0061 (14)	−0.0010 (14)	−0.0069 (14)
C17	0.065 (2)	0.0602 (19)	0.0492 (19)	0.0174 (16)	0.0070 (15)	−0.0057 (16)
C18	0.094 (3)	0.082 (2)	0.064 (2)	0.043 (2)	0.020 (2)	0.018 (2)
C19	0.0326 (13)	0.0353 (13)	0.0455 (15)	0.0043 (10)	−0.0001 (10)	0.0017 (11)
C20	0.0500 (17)	0.0435 (15)	0.0571 (18)	0.0035 (13)	0.0064 (13)	0.0143 (13)
C21	0.0540 (18)	0.0390 (14)	0.079 (2)	0.0129 (14)	−0.0166 (16)	−0.0118 (15)
C22A	0.027 (5)	0.068 (7)	0.037 (6)	−0.009 (4)	0.000 (3)	0.007 (5)
C23A	0.092 (9)	0.110 (8)	0.060 (6)	0.011 (7)	0.044 (5)	0.004 (6)
C22B	0.049 (7)	0.075 (7)	0.039 (5)	−0.007 (4)	−0.001 (4)	0.008 (4)
C23B	0.043 (4)	0.139 (8)	0.071 (5)	0.006 (5)	0.028 (4)	0.041 (6)
C24	0.064 (2)	0.087 (3)	0.057 (2)	−0.013 (2)	−0.0065 (17)	0.0274 (19)
C25	0.0415 (14)	0.0334 (13)	0.068 (2)	−0.0068 (11)	0.0038 (14)	0.0027 (13)
C26	0.066 (2)	0.0347 (15)	0.103 (3)	0.0021 (15)	0.005 (2)	0.0005 (17)
C27	0.0453 (17)	0.0563 (18)	0.089 (3)	−0.0126 (15)	−0.0059 (17)	−0.0042 (18)
C28	0.0494 (15)	0.0377 (13)	0.0424 (15)	0.0023 (12)	−0.0056 (12)	0.0025 (11)
C29	0.097 (3)	0.0343 (13)	0.0561 (19)	−0.0033 (17)	−0.0090 (17)	0.0018 (14)
C39	0.070 (2)	0.0478 (15)	0.0417 (16)	0.0058 (15)	−0.0047 (14)	0.0052 (13)
C31	0.0330 (13)	0.0482 (14)	0.0367 (14)	−0.0009 (12)	0.0000 (10)	−0.0029 (11)
C32	0.065 (2)	0.071 (2)	0.0370 (15)	0.0091 (17)	0.0065 (13)	0.0109 (15)
C33	0.0461 (18)	0.103 (3)	0.053 (2)	0.0208 (18)	0.0082 (14)	−0.0075 (19)
C34	0.0441 (17)	0.065 (2)	0.080 (3)	−0.0193 (15)	−0.0043 (16)	−0.0158 (18)
C35	0.0390 (14)	0.0372 (13)	0.0384 (14)	−0.0075 (11)	−0.0053 (10)	−0.0041 (11)
C36	0.084 (2)	0.0353 (14)	0.065 (2)	−0.0111 (14)	−0.0190 (19)	0.0013 (14)

### Methyl (R)-3-{(1R,4S)-6-methoxy-4,7-dimethyl-5,8-bis[(triisopropylsilyl)oxy]-1,2,3,4-tetrahydronaphthalen-1-yl}butanoate (2) Geometric parameters (Å, º)

**Table:**

Si1—O1	1.6706 (17)	C19—C21	1.531 (4)
Si1—C22B	1.860 (13)	C19—H19	1.0000
Si1—C25	1.873 (3)	C20—H20A	0.9800
Si1—C19	1.886 (3)	C20—H20B	0.9800
Si1—C22A	1.949 (14)	C20—H20C	0.9800
Si2—O3	1.6689 (17)	C21—H21A	0.9800
Si2—C35	1.880 (3)	C21—H21B	0.9800
Si2—C31	1.883 (3)	C21—H21C	0.9800
Si2—C28	1.883 (3)	C22A—C23A	1.265 (18)
O1—C1	1.374 (3)	C22A—C24	1.511 (15)
O2—C2	1.387 (3)	C22A—H22A	1.0000
O2—C13	1.420 (4)	C23A—H23A	0.9800
O3—C4	1.381 (3)	C23A—H23B	0.9800
O5—C17	1.326 (4)	C23A—H23C	0.9800
O5—C18	1.453 (4)	C22B—C23B	1.344 (15)
O4A—O4B	0.94 (4)	C22B—C24	1.555 (13)
O4A—C17	1.1977 (13)	C22B—H22B	1.0000
O4B—C17	1.1960 (14)	C23B—H23D	0.9800
C1—C2	1.381 (3)	C23B—H23E	0.9800
C1—C10	1.408 (3)	C23B—H23F	0.9800
C2—C3	1.391 (3)	C24—H24A	0.9800
C3—C4	1.396 (3)	C24—H24B	0.9800
C3—C12	1.509 (3)	C24—H24C	0.9800
C4—C5	1.408 (3)	C25—C26	1.535 (4)
C5—C10	1.387 (3)	C25—C27	1.537 (4)
C5—C6	1.530 (3)	C25—H25	1.0000
C6—C7	1.534 (4)	C26—H26A	0.9800
C6—C14	1.538 (4)	C26—H26B	0.9800
C6—H6	1.0000	C26—H26C	0.9800
C7—C8	1.518 (4)	C27—H27A	0.9800
C7—H7A	0.9900	C27—H27B	0.9800
C7—H7B	0.9900	C27—H27C	0.9800
C8—C9	1.524 (4)	C28—C39	1.515 (4)
C8—H8A	0.9900	C28—C29	1.523 (4)
C8—H8B	0.9900	C28—H28	1.0000
C9—C10	1.524 (3)	C29—H29A	0.9800
C9—C11	1.535 (4)	C29—H29B	0.9800
C9—H9	1.0000	C29—H29C	0.9800
C11—H11A	0.9800	C39—H39A	0.9800
C11—H11B	0.9800	C39—H39B	0.9800
C11—H11C	0.9800	C39—H39C	0.9800
C12—H12A	0.9800	C31—C32	1.525 (4)
C12—H12B	0.9800	C31—C33	1.530 (4)
C12—H12C	0.9800	C31—H31	1.0000
C13—H13A	0.9800	C32—H32A	0.9800
C13—H13B	0.9800	C32—H32B	0.9800
C13—H13C	0.9800	C32—H32C	0.9800
C14—C16	1.524 (4)	C33—H33A	0.9800
C14—C15	1.535 (4)	C33—H33B	0.9800
C14—H14	1.0000	C33—H33C	0.9800
C15—H15A	0.9800	C34—C35	1.531 (4)
C15—H15B	0.9800	C34—H34A	0.9800
C15—H15C	0.9800	C34—H34B	0.9800
C16—C17	1.504 (4)	C34—H34C	0.9800
C16—H16A	0.9900	C35—C36	1.532 (4)
C16—H16B	0.9900	C35—H35	1.0000
C18—H18A	0.9800	C36—H36A	0.9800
C18—H18B	0.9800	C36—H36B	0.9800
C18—H18C	0.9800	C36—H36C	0.9800
C19—C20	1.529 (4)		
			
O1—Si1—C22B	110.2 (4)	Si1—C19—H19	105.9
O1—Si1—C25	108.63 (12)	C19—C20—H20A	109.5
C22B—Si1—C25	104.8 (4)	C19—C20—H20B	109.5
O1—Si1—C19	111.64 (11)	H20A—C20—H20B	109.5
C22B—Si1—C19	110.9 (4)	C19—C20—H20C	109.5
C25—Si1—C19	110.34 (13)	H20A—C20—H20C	109.5
O1—Si1—C22A	98.5 (4)	H20B—C20—H20C	109.5
C25—Si1—C22A	120.5 (4)	C19—C21—H21A	109.5
C19—Si1—C22A	106.7 (5)	C19—C21—H21B	109.5
O3—Si2—C35	104.10 (10)	H21A—C21—H21B	109.5
O3—Si2—C31	107.32 (11)	C19—C21—H21C	109.5
C35—Si2—C31	114.84 (12)	H21A—C21—H21C	109.5
O3—Si2—C28	113.89 (12)	H21B—C21—H21C	109.5
C35—Si2—C28	109.38 (12)	C23A—C22A—C24	124.2 (11)
C31—Si2—C28	107.46 (12)	C23A—C22A—Si1	124.5 (10)
C1—O1—Si1	131.04 (16)	C24—C22A—Si1	111.0 (8)
C2—O2—C13	112.7 (2)	C23A—C22A—H22A	91.7
C4—O3—Si2	131.97 (15)	C24—C22A—H22A	91.7
C17—O5—C18	115.0 (3)	Si1—C22A—H22A	91.7
O4B—O4A—C17	66.7 (10)	C22A—C23A—H23A	109.5
O4A—O4B—C17	66.9 (10)	C22A—C23A—H23B	109.5
O1—C1—C2	120.5 (2)	H23A—C23A—H23B	109.5
O1—C1—C10	119.6 (2)	C22A—C23A—H23C	109.5
C2—C1—C10	119.8 (2)	H23A—C23A—H23C	109.5
C1—C2—O2	118.7 (2)	H23B—C23A—H23C	109.5
C1—C2—C3	121.8 (2)	C23B—C22B—C24	120.0 (9)
O2—C2—C3	119.4 (2)	C23B—C22B—Si1	126.3 (9)
C2—C3—C4	117.7 (2)	C24—C22B—Si1	113.6 (8)
C2—C3—C12	120.4 (2)	C23B—C22B—H22B	90.1
C4—C3—C12	121.8 (2)	C24—C22B—H22B	90.1
O3—C4—C3	118.7 (2)	Si1—C22B—H22B	90.1
O3—C4—C5	119.6 (2)	C22B—C23B—H23D	109.5
C3—C4—C5	121.7 (2)	C22B—C23B—H23E	109.5
C10—C5—C4	118.8 (2)	H23D—C23B—H23E	109.5
C10—C5—C6	122.3 (2)	C22B—C23B—H23F	109.5
C4—C5—C6	118.9 (2)	H23D—C23B—H23F	109.5
C5—C6—C7	111.8 (2)	H23E—C23B—H23F	109.5
C5—C6—C14	111.3 (2)	C22A—C24—H24A	109.5
C7—C6—C14	115.4 (2)	C22A—C24—H24B	109.5
C5—C6—H6	105.9	H24A—C24—H24B	109.5
C7—C6—H6	105.9	C22A—C24—H24C	109.5
C14—C6—H6	105.9	H24A—C24—H24C	109.5
C8—C7—C6	113.9 (2)	H24B—C24—H24C	109.5
C8—C7—H7A	108.8	C26—C25—C27	109.8 (2)
C6—C7—H7A	108.8	C26—C25—Si1	114.6 (2)
C8—C7—H7B	108.8	C27—C25—Si1	113.6 (2)
C6—C7—H7B	108.8	C26—C25—H25	106.1
H7A—C7—H7B	107.7	C27—C25—H25	106.1
C7—C8—C9	111.8 (2)	Si1—C25—H25	106.1
C7—C8—H8A	109.3	C25—C26—H26A	109.5
C9—C8—H8A	109.3	C25—C26—H26B	109.5
C7—C8—H8B	109.3	H26A—C26—H26B	109.5
C9—C8—H8B	109.3	C25—C26—H26C	109.5
H8A—C8—H8B	107.9	H26A—C26—H26C	109.5
C10—C9—C8	111.8 (2)	H26B—C26—H26C	109.5
C10—C9—C11	108.5 (2)	C25—C27—H27A	109.5
C8—C9—C11	112.1 (2)	C25—C27—H27B	109.5
C10—C9—H9	108.1	H27A—C27—H27B	109.5
C8—C9—H9	108.1	C25—C27—H27C	109.5
C11—C9—H9	108.1	H27A—C27—H27C	109.5
C5—C10—C1	119.9 (2)	H27B—C27—H27C	109.5
C5—C10—C9	122.4 (2)	C39—C28—C29	110.3 (3)
C1—C10—C9	117.6 (2)	C39—C28—Si2	116.4 (2)
C9—C11—H11A	109.5	C29—C28—Si2	113.5 (2)
C9—C11—H11B	109.5	C39—C28—H28	105.2
H11A—C11—H11B	109.5	C29—C28—H28	105.2
C9—C11—H11C	109.5	Si2—C28—H28	105.2
H11A—C11—H11C	109.5	C28—C29—H29A	109.5
H11B—C11—H11C	109.5	C28—C29—H29B	109.5
C3—C12—H12A	109.5	H29A—C29—H29B	109.5
C3—C12—H12B	109.5	C28—C29—H29C	109.5
H12A—C12—H12B	109.5	H29A—C29—H29C	109.5
C3—C12—H12C	109.5	H29B—C29—H29C	109.5
H12A—C12—H12C	109.5	C28—C39—H39A	109.5
H12B—C12—H12C	109.5	C28—C39—H39B	109.5
O2—C13—H13A	109.5	H39A—C39—H39B	109.5
O2—C13—H13B	109.5	C28—C39—H39C	109.5
H13A—C13—H13B	109.5	H39A—C39—H39C	109.5
O2—C13—H13C	109.5	H39B—C39—H39C	109.5
H13A—C13—H13C	109.5	C32—C31—C33	109.6 (3)
H13B—C13—H13C	109.5	C32—C31—Si2	114.43 (19)
C16—C14—C15	109.5 (3)	C33—C31—Si2	115.1 (2)
C16—C14—C6	114.9 (2)	C32—C31—H31	105.6
C15—C14—C6	111.9 (3)	C33—C31—H31	105.6
C16—C14—H14	106.7	Si2—C31—H31	105.6
C15—C14—H14	106.7	C31—C32—H32A	109.5
C6—C14—H14	106.7	C31—C32—H32B	109.5
C14—C15—H15A	109.5	H32A—C32—H32B	109.5
C14—C15—H15B	109.5	C31—C32—H32C	109.5
H15A—C15—H15B	109.5	H32A—C32—H32C	109.5
C14—C15—H15C	109.5	H32B—C32—H32C	109.5
H15A—C15—H15C	109.5	C31—C33—H33A	109.5
H15B—C15—H15C	109.5	C31—C33—H33B	109.5
C17—C16—C14	111.7 (3)	H33A—C33—H33B	109.5
C17—C16—H16A	109.3	C31—C33—H33C	109.5
C14—C16—H16A	109.3	H33A—C33—H33C	109.5
C17—C16—H16B	109.3	H33B—C33—H33C	109.5
C14—C16—H16B	109.3	C35—C34—H34A	109.5
H16A—C16—H16B	107.9	C35—C34—H34B	109.5
O4B—C17—O4A	46 (2)	H34A—C34—H34B	109.5
O4B—C17—O5	108.1 (17)	C35—C34—H34C	109.5
O4A—C17—O5	122.4 (3)	H34A—C34—H34C	109.5
O4B—C17—C16	120.4 (18)	H34B—C34—H34C	109.5
O4A—C17—C16	125.9 (3)	C34—C35—C36	110.5 (3)
O5—C17—C16	111.5 (2)	C34—C35—Si2	113.2 (2)
O5—C18—H18A	109.5	C36—C35—Si2	114.49 (19)
O5—C18—H18B	109.5	C34—C35—H35	106.0
H18A—C18—H18B	109.5	C36—C35—H35	106.0
O5—C18—H18C	109.5	Si2—C35—H35	106.0
H18A—C18—H18C	109.5	C35—C36—H36A	109.5
H18B—C18—H18C	109.5	C35—C36—H36B	109.5
C20—C19—C21	110.2 (2)	H36A—C36—H36B	109.5
C20—C19—Si1	114.52 (19)	C35—C36—H36C	109.5
C21—C19—Si1	113.55 (19)	H36A—C36—H36C	109.5
C20—C19—H19	105.9	H36B—C36—H36C	109.5
C21—C19—H19	105.9		

### Methyl (R)-3-{(1R,4S)-6-methoxy-4,7-dimethyl-5,8-bis[(triisopropylsilyl)oxy]-1,2,3,4-tetrahydronaphthalen-1-yl}butanoate (2) Hydrogen-bond geometry (Å, º)

**Table:**

*D*—H···*A*	*D*—H	H···*A*	*D*···*A*	*D*—H···*A*
C14—H14···O3	1.00	2.46	3.115 (3)	123
C25—H25···O4*B*^i^	1.00	2.46	3.312 (13)	142

Symmetry code: (i) −*x*+1, *y*−1/2, −*z*+1/2.

### Methyl (E)-3-{(1R,4S)-8-hydroxy-6-methoxy-4,7-dimethyl-5-[(triisopropylsilyl)oxy]-1,2,3,4-tetrahydronaphthalen-1-yl}acrylate (8) Crystal data

**Table:**

C_26_H_42_O_5_Si	*F*(000) = 504
*M**_r_* = 462.68	*D*_x_ = 1.173 Mg m^−^^3^
Monoclinic, *P*2_1_	Mo *K*α radiation, λ = 0.71073 Å
*a* = 12.0078 (7) Å	Cell parameters from 9955 reflections
*b* = 9.2620 (6) Å	θ = 2.7–35.2°
*c* = 12.1411 (8) Å	µ = 0.12 mm^−^^1^
β = 104.0912 (14)°	*T* = 100 K
*V* = 1309.66 (14) Å^3^	Fragment, colourless
*Z* = 2	0.6 × 0.5 × 0.4 mm

### Methyl (E)-3-{(1R,4S)-8-hydroxy-6-methoxy-4,7-dimethyl-5-[(triisopropylsilyl)oxy]-1,2,3,4-tetrahydronaphthalen-1-yl}acrylate (8) Data collection

**Table:**

Bruker APEXII CCD diffractometer	10749 reflections with *I* > 2σ(*I*)
ω–scans	*R*_int_ = 0.028
Absorption correction: multi-scan (*SADABS*; Krause *et al.*, 2015)	θ_max_ = 35.4°, θ_min_ = 1.7°
*T*_min_ = 0.672, *T*_max_ = 0.747	*h* = −19→19
39668 measured reflections	*k* = −15→14
11727 independent reflections	*l* = −19→19

### Methyl (E)-3-{(1R,4S)-8-hydroxy-6-methoxy-4,7-dimethyl-5-[(triisopropylsilyl)oxy]-1,2,3,4-tetrahydronaphthalen-1-yl}acrylate (8) Refinement

**Table:**

Refinement on *F*^2^	Hydrogen site location: mixed
Least-squares matrix: full	H atoms treated by a mixture of independent and constrained refinement
*R*[*F*^2^ > 2σ(*F*^2^)] = 0.035	*w* = 1/[σ^2^(*F*_o_^2^) + (0.0515*P*)^2^ + 0.0894*P*] where *P* = (*F*_o_^2^ + 2*F*_c_^2^)/3
*wR*(*F*^2^) = 0.088	(Δ/σ)_max_ < 0.001
*S* = 1.04	Δρ_max_ = 0.47 e Å^−^^3^
11727 reflections	Δρ_min_ = −0.18 e Å^−^^3^
303 parameters	Absolute structure: Flack *x* determined using 4647 quotients [(*I*^+^)-(*I*^-^)]/[(*I*^+^)+(*I*^-^)] (Parsons *et al.*, 2013)
1 restraint	Absolute structure parameter: 0.02 (2)

### Methyl (E)-3-{(1R,4S)-8-hydroxy-6-methoxy-4,7-dimethyl-5-[(triisopropylsilyl)oxy]-1,2,3,4-tetrahydronaphthalen-1-yl}acrylate (8) Special details

**Table:**

Geometry. All esds (except the esd in the dihedral angle between two l.s. planes) are estimated using the full covariance matrix. The cell esds are taken into account individually in the estimation of esds in distances, angles and torsion angles; correlations between esds in cell parameters are only used when they are defined by crystal symmetry. An approximate (isotropic) treatment of cell esds is used for estimating esds involving l.s. planes.

### Methyl (E)-3-{(1R,4S)-8-hydroxy-6-methoxy-4,7-dimethyl-5-[(triisopropylsilyl)oxy]-1,2,3,4-tetrahydronaphthalen-1-yl}acrylate (8) Fractional atomic coordinates and isotropic or equivalent isotropic displacement parameters (Å^2^)

**Table:**

	*x*	*y*	*z*	*U*_iso_*/*U*_eq_	
Si1	0.48053 (3)	0.48209 (4)	0.75923 (3)	0.01557 (7)	
O1	0.36877 (7)	0.39726 (10)	0.67418 (7)	0.01631 (15)	
O2	0.34040 (8)	0.16094 (10)	0.80383 (8)	0.01990 (17)	
O3	−0.05396 (7)	0.29748 (11)	0.76559 (8)	0.01824 (16)	
O4	0.02497 (9)	0.76919 (12)	0.99403 (8)	0.0249 (2)	
O5	−0.12137 (8)	0.83904 (11)	0.85055 (8)	0.02317 (19)	
C1	0.26232 (9)	0.36874 (12)	0.69351 (9)	0.01257 (17)	
C2	0.24784 (9)	0.25016 (12)	0.75946 (9)	0.01384 (18)	
C3	0.14194 (10)	0.22030 (12)	0.78344 (9)	0.01415 (18)	
C4	0.05190 (9)	0.31694 (12)	0.74235 (9)	0.01276 (17)	
C5	0.06246 (9)	0.43187 (11)	0.67032 (8)	0.01160 (17)	
C6	−0.04152 (9)	0.52688 (12)	0.62464 (9)	0.01299 (17)	
H6	−0.109842	0.461932	0.602372	0.016*	
C7	−0.03128 (10)	0.60915 (14)	0.51721 (10)	0.0177 (2)	
H7A	−0.040547	0.541030	0.452773	0.021*	
H7B	−0.092819	0.682593	0.497381	0.021*	
C8	0.08556 (10)	0.68242 (13)	0.53806 (10)	0.0185 (2)	
H8A	0.095731	0.747190	0.604642	0.022*	
H8B	0.088818	0.742123	0.471282	0.022*	
C9	0.18316 (10)	0.57161 (13)	0.55933 (10)	0.01534 (18)	
H9	0.256473	0.623814	0.592855	0.018*	
C10	0.16797 (9)	0.45609 (11)	0.64295 (8)	0.01165 (17)	
C11	0.19429 (12)	0.50138 (16)	0.44744 (10)	0.0236 (2)	
H11A	0.123449	0.449089	0.412895	0.035*	
H11B	0.259098	0.433894	0.463107	0.035*	
H11C	0.207407	0.576506	0.395167	0.035*	
C12	0.12435 (12)	0.08650 (15)	0.84750 (12)	0.0225 (2)	
H12A	0.046266	0.049968	0.817383	0.034*	
H12B	0.135394	0.109803	0.928181	0.034*	
H12C	0.179941	0.012606	0.838658	0.034*	
C13	0.36061 (15)	0.06106 (18)	0.72106 (14)	0.0319 (3)	
H13A	0.370301	0.114056	0.654150	0.048*	
H13B	0.295071	−0.004772	0.698876	0.048*	
H13C	0.430319	0.005408	0.753395	0.048*	
C14	−0.06518 (10)	0.63272 (12)	0.71045 (10)	0.01513 (18)	
H14	−0.133258	0.688688	0.688128	0.018*	
C15	0.00034 (10)	0.65543 (14)	0.81490 (10)	0.0168 (2)	
H15	0.069598	0.601995	0.838431	0.020*	
C16	−0.02911 (10)	0.75888 (13)	0.89544 (10)	0.0170 (2)	
C17	−0.15650 (15)	0.93869 (17)	0.92681 (14)	0.0300 (3)	
H17A	−0.096621	1.011732	0.951783	0.045*	
H17B	−0.168588	0.886115	0.992980	0.045*	
H17C	−0.228183	0.985992	0.887521	0.045*	
C18	0.44412 (11)	0.56553 (15)	0.88786 (11)	0.0201 (2)	
H18	0.511801	0.626535	0.924246	0.024*	
C19	0.42985 (14)	0.45753 (19)	0.97945 (12)	0.0293 (3)	
H19A	0.364137	0.394431	0.948636	0.044*	
H19B	0.416689	0.510226	1.045134	0.044*	
H19C	0.499609	0.399038	1.003030	0.044*	
C20	0.34128 (13)	0.66911 (19)	0.85885 (15)	0.0312 (3)	
H20A	0.333252	0.717962	0.928104	0.047*	
H20B	0.271195	0.614413	0.826038	0.047*	
H20C	0.353758	0.741075	0.803905	0.047*	
C21	0.60109 (15)	0.7456 (2)	0.73612 (16)	0.0340 (3)	
H21A	0.607704	0.826546	0.686133	0.051*	
H21B	0.676685	0.701172	0.764775	0.051*	
H21C	0.572320	0.780852	0.800116	0.051*	
C22	0.51751 (12)	0.63325 (18)	0.66939 (12)	0.0271 (3)	
H22	0.444112	0.685691	0.636660	0.032*	
C23	0.5620 (2)	0.5802 (3)	0.56774 (16)	0.0545 (6)	
H23A	0.638360	0.537435	0.595334	0.082*	
H23B	0.566687	0.661862	0.517766	0.082*	
H23C	0.509220	0.507466	0.525434	0.082*	
C24	0.59760 (11)	0.34486 (17)	0.80858 (11)	0.0235 (2)	
H24	0.567061	0.274892	0.856690	0.028*	
C25	0.62999 (17)	0.2544 (3)	0.71550 (17)	0.0449 (5)	
H25A	0.677701	0.172712	0.750081	0.067*	
H25B	0.672941	0.314393	0.673627	0.067*	
H25C	0.560063	0.218341	0.663202	0.067*	
C26	0.70560 (12)	0.4103 (2)	0.88641 (15)	0.0354 (4)	
H26A	0.743452	0.473543	0.841963	0.053*	
H26B	0.758258	0.332748	0.920602	0.053*	
H26C	0.684181	0.466512	0.946489	0.053*	
H1	−0.0445 (18)	0.280 (3)	0.8345 (18)	0.032 (5)*	

### Methyl (E)-3-{(1R,4S)-8-hydroxy-6-methoxy-4,7-dimethyl-5-[(triisopropylsilyl)oxy]-1,2,3,4-tetrahydronaphthalen-1-yl}acrylate (8) Atomic displacement parameters (Å^2^)

**Table:**

	*U*^11^	*U*^22^	*U*^33^	*U*^12^	*U*^13^	*U*^23^
Si1	0.01057 (12)	0.02079 (15)	0.01515 (13)	−0.00063 (11)	0.00275 (9)	0.00242 (12)
O1	0.0112 (3)	0.0211 (4)	0.0165 (3)	−0.0007 (3)	0.0031 (3)	0.0012 (3)
O2	0.0174 (4)	0.0202 (4)	0.0206 (4)	0.0071 (3)	0.0018 (3)	0.0056 (3)
O3	0.0139 (3)	0.0254 (4)	0.0160 (4)	−0.0009 (3)	0.0048 (3)	0.0039 (3)
O4	0.0253 (4)	0.0334 (5)	0.0154 (4)	−0.0011 (4)	0.0037 (3)	−0.0063 (4)
O5	0.0221 (4)	0.0229 (4)	0.0235 (4)	0.0050 (4)	0.0037 (3)	−0.0082 (4)
C1	0.0107 (4)	0.0139 (4)	0.0125 (4)	−0.0004 (3)	0.0016 (3)	0.0007 (3)
C2	0.0132 (4)	0.0141 (4)	0.0132 (4)	0.0024 (3)	0.0012 (3)	0.0025 (3)
C3	0.0154 (4)	0.0132 (4)	0.0133 (4)	0.0004 (3)	0.0024 (3)	0.0029 (3)
C4	0.0131 (4)	0.0134 (4)	0.0114 (4)	−0.0008 (3)	0.0021 (3)	0.0009 (3)
C5	0.0111 (4)	0.0124 (4)	0.0102 (4)	0.0002 (3)	0.0004 (3)	−0.0003 (3)
C6	0.0123 (4)	0.0140 (4)	0.0113 (4)	0.0013 (3)	0.0002 (3)	−0.0006 (3)
C7	0.0170 (5)	0.0199 (5)	0.0140 (4)	0.0038 (4)	−0.0003 (3)	0.0032 (4)
C8	0.0199 (5)	0.0146 (5)	0.0198 (5)	0.0016 (4)	0.0029 (4)	0.0055 (4)
C9	0.0151 (4)	0.0149 (4)	0.0153 (4)	−0.0008 (4)	0.0023 (3)	0.0043 (4)
C10	0.0119 (4)	0.0113 (4)	0.0108 (4)	−0.0006 (3)	0.0010 (3)	0.0008 (3)
C11	0.0284 (6)	0.0276 (6)	0.0170 (5)	0.0030 (5)	0.0099 (4)	0.0049 (4)
C12	0.0238 (6)	0.0195 (5)	0.0254 (6)	0.0014 (4)	0.0084 (5)	0.0107 (4)
C13	0.0372 (8)	0.0264 (7)	0.0338 (7)	0.0162 (6)	0.0120 (6)	0.0029 (6)
C14	0.0142 (4)	0.0151 (5)	0.0152 (4)	0.0015 (4)	0.0018 (3)	−0.0005 (4)
C15	0.0148 (4)	0.0195 (5)	0.0155 (4)	0.0018 (4)	0.0022 (4)	−0.0029 (4)
C16	0.0156 (4)	0.0192 (5)	0.0169 (4)	−0.0027 (4)	0.0056 (4)	−0.0031 (4)
C17	0.0348 (7)	0.0251 (6)	0.0334 (7)	0.0057 (5)	0.0145 (6)	−0.0088 (5)
C18	0.0162 (5)	0.0231 (5)	0.0212 (5)	−0.0030 (4)	0.0046 (4)	−0.0028 (4)
C19	0.0346 (7)	0.0359 (8)	0.0207 (5)	−0.0062 (6)	0.0130 (5)	−0.0034 (5)
C20	0.0242 (6)	0.0309 (7)	0.0368 (7)	0.0032 (5)	0.0045 (5)	−0.0135 (6)
C21	0.0305 (7)	0.0325 (8)	0.0400 (8)	−0.0133 (6)	0.0105 (6)	0.0024 (6)
C22	0.0190 (5)	0.0347 (7)	0.0262 (6)	−0.0081 (5)	0.0029 (4)	0.0102 (5)
C23	0.0761 (15)	0.0658 (14)	0.0279 (8)	−0.0325 (12)	0.0252 (9)	−0.0012 (9)
C24	0.0143 (5)	0.0329 (7)	0.0223 (5)	0.0058 (5)	0.0024 (4)	0.0028 (5)
C25	0.0346 (8)	0.0584 (12)	0.0415 (9)	0.0232 (8)	0.0090 (7)	−0.0081 (9)
C26	0.0147 (5)	0.0514 (10)	0.0351 (8)	0.0021 (6)	−0.0035 (5)	0.0069 (7)

### Methyl (E)-3-{(1R,4S)-8-hydroxy-6-methoxy-4,7-dimethyl-5-[(triisopropylsilyl)oxy]-1,2,3,4-tetrahydronaphthalen-1-yl}acrylate (8) Geometric parameters (Å, º)

**Table:**

Si1—O1	1.6763 (9)	C13—H13A	0.9800
Si1—C24	1.8816 (14)	C13—H13B	0.9800
Si1—C18	1.8867 (13)	C13—H13C	0.9800
Si1—C22	1.8928 (14)	C14—C15	1.3363 (16)
O1—C1	1.3802 (13)	C14—H14	0.9500
O2—C2	1.3845 (14)	C15—C16	1.4723 (16)
O2—C13	1.4295 (18)	C15—H15	0.9500
O3—C4	1.3793 (13)	C17—H17A	0.9800
O3—H1	0.83 (2)	C17—H17B	0.9800
O4—C16	1.2186 (15)	C17—H17C	0.9800
O5—C16	1.3348 (15)	C18—C20	1.535 (2)
O5—C17	1.4413 (16)	C18—C19	1.536 (2)
C1—C2	1.3947 (15)	C18—H18	1.0000
C1—C10	1.4058 (14)	C19—H19A	0.9800
C2—C3	1.3994 (15)	C19—H19B	0.9800
C3—C4	1.3987 (15)	C19—H19C	0.9800
C3—C12	1.5051 (17)	C20—H20A	0.9800
C4—C5	1.4029 (15)	C20—H20B	0.9800
C5—C10	1.4039 (15)	C20—H20C	0.9800
C5—C6	1.5177 (15)	C21—C22	1.533 (2)
C6—C14	1.5070 (15)	C21—H21A	0.9800
C6—C7	1.5411 (16)	C21—H21B	0.9800
C6—H6	1.0000	C21—H21C	0.9800
C7—C8	1.5233 (18)	C22—C23	1.540 (3)
C7—H7A	0.9900	C22—H22	1.0000
C7—H7B	0.9900	C23—H23A	0.9800
C8—C9	1.5317 (16)	C23—H23B	0.9800
C8—H8A	0.9900	C23—H23C	0.9800
C8—H8B	0.9900	C24—C25	1.531 (2)
C9—C10	1.5162 (15)	C24—C26	1.531 (2)
C9—C11	1.5410 (17)	C24—H24	1.0000
C9—H9	1.0000	C25—H25A	0.9800
C11—H11A	0.9800	C25—H25B	0.9800
C11—H11B	0.9800	C25—H25C	0.9800
C11—H11C	0.9800	C26—H26A	0.9800
C12—H12A	0.9800	C26—H26B	0.9800
C12—H12B	0.9800	C26—H26C	0.9800
C12—H12C	0.9800		
			
O1—Si1—C24	107.67 (6)	C15—C14—C6	126.29 (10)
O1—Si1—C18	112.94 (5)	C15—C14—H14	116.9
C24—Si1—C18	108.59 (6)	C6—C14—H14	116.9
O1—Si1—C22	104.87 (5)	C14—C15—C16	123.48 (11)
C24—Si1—C22	114.91 (7)	C14—C15—H15	118.3
C18—Si1—C22	107.95 (7)	C16—C15—H15	118.3
C1—O1—Si1	128.37 (7)	O4—C16—O5	123.25 (11)
C2—O2—C13	111.74 (10)	O4—C16—C15	123.14 (11)
C4—O3—H1	108.9 (14)	O5—C16—C15	113.60 (10)
C16—O5—C17	115.95 (11)	O5—C17—H17A	109.5
O1—C1—C2	119.89 (9)	O5—C17—H17B	109.5
O1—C1—C10	119.79 (9)	H17A—C17—H17B	109.5
C2—C1—C10	120.27 (9)	O5—C17—H17C	109.5
O2—C2—C1	119.53 (10)	H17A—C17—H17C	109.5
O2—C2—C3	119.02 (10)	H17B—C17—H17C	109.5
C1—C2—C3	121.44 (10)	C20—C18—C19	110.34 (12)
C4—C3—C2	117.51 (10)	C20—C18—Si1	113.68 (10)
C4—C3—C12	121.17 (10)	C19—C18—Si1	114.94 (10)
C2—C3—C12	121.28 (10)	C20—C18—H18	105.7
O3—C4—C3	120.95 (10)	C19—C18—H18	105.7
O3—C4—C5	117.02 (9)	Si1—C18—H18	105.7
C3—C4—C5	121.91 (10)	C18—C19—H19A	109.5
C4—C5—C10	119.51 (9)	C18—C19—H19B	109.5
C4—C5—C6	118.46 (9)	H19A—C19—H19B	109.5
C10—C5—C6	122.03 (9)	C18—C19—H19C	109.5
C14—C6—C5	113.66 (9)	H19A—C19—H19C	109.5
C14—C6—C7	109.32 (9)	H19B—C19—H19C	109.5
C5—C6—C7	111.58 (9)	C18—C20—H20A	109.5
C14—C6—H6	107.3	C18—C20—H20B	109.5
C5—C6—H6	107.3	H20A—C20—H20B	109.5
C7—C6—H6	107.3	C18—C20—H20C	109.5
C8—C7—C6	109.86 (9)	H20A—C20—H20C	109.5
C8—C7—H7A	109.7	H20B—C20—H20C	109.5
C6—C7—H7A	109.7	C22—C21—H21A	109.5
C8—C7—H7B	109.7	C22—C21—H21B	109.5
C6—C7—H7B	109.7	H21A—C21—H21B	109.5
H7A—C7—H7B	108.2	C22—C21—H21C	109.5
C7—C8—C9	111.43 (10)	H21A—C21—H21C	109.5
C7—C8—H8A	109.3	H21B—C21—H21C	109.5
C9—C8—H8A	109.3	C21—C22—C23	109.27 (14)
C7—C8—H8B	109.3	C21—C22—Si1	114.38 (10)
C9—C8—H8B	109.3	C23—C22—Si1	113.67 (13)
H8A—C8—H8B	108.0	C21—C22—H22	106.3
C10—C9—C8	111.96 (9)	C23—C22—H22	106.3
C10—C9—C11	110.05 (10)	Si1—C22—H22	106.3
C8—C9—C11	111.18 (10)	C22—C23—H23A	109.5
C10—C9—H9	107.8	C22—C23—H23B	109.5
C8—C9—H9	107.8	H23A—C23—H23B	109.5
C11—C9—H9	107.8	C22—C23—H23C	109.5
C5—C10—C1	118.84 (9)	H23A—C23—H23C	109.5
C5—C10—C9	122.27 (9)	H23B—C23—H23C	109.5
C1—C10—C9	118.88 (9)	C25—C24—C26	110.06 (13)
C9—C11—H11A	109.5	C25—C24—Si1	116.03 (10)
C9—C11—H11B	109.5	C26—C24—Si1	112.57 (12)
H11A—C11—H11B	109.5	C25—C24—H24	105.8
C9—C11—H11C	109.5	C26—C24—H24	105.8
H11A—C11—H11C	109.5	Si1—C24—H24	105.8
H11B—C11—H11C	109.5	C24—C25—H25A	109.5
C3—C12—H12A	109.5	C24—C25—H25B	109.5
C3—C12—H12B	109.5	H25A—C25—H25B	109.5
H12A—C12—H12B	109.5	C24—C25—H25C	109.5
C3—C12—H12C	109.5	H25A—C25—H25C	109.5
H12A—C12—H12C	109.5	H25B—C25—H25C	109.5
H12B—C12—H12C	109.5	C24—C26—H26A	109.5
O2—C13—H13A	109.5	C24—C26—H26B	109.5
O2—C13—H13B	109.5	H26A—C26—H26B	109.5
H13A—C13—H13B	109.5	C24—C26—H26C	109.5
O2—C13—H13C	109.5	H26A—C26—H26C	109.5
H13A—C13—H13C	109.5	H26B—C26—H26C	109.5
H13B—C13—H13C	109.5		

### Methyl (E)-3-{(1R,4S)-8-hydroxy-6-methoxy-4,7-dimethyl-5-[(triisopropylsilyl)oxy]-1,2,3,4-tetrahydronaphthalen-1-yl}acrylate (8) Hydrogen-bond geometry (Å, º)

**Table:**

*D*—H···*A*	*D*—H	H···*A*	*D*···*A*	*D*—H···*A*
O3—H1···O4^i^	0.83 (2)	2.04 (2)	2.8658 (13)	171 (2)
C17—H17*A*···O4^ii^	0.98	2.57	3.471 (2)	154

Symmetry codes: (i) −*x*, *y*−1/2, −*z*+2; (ii) −*x*, *y*+1/2, −*z*+2.
