# Supplementary material for: Crystal structures of two 1,2,3,4-tetra­hydro­naphthalenes obtained during efforts towards the total synthesis of elisabethin A
Source: Acta Crystallogr E Crystallogr Commun. 2023 Feb 17;79(Pt 3):177–81. doi: 10.1107/S2056989023001226 (PMC9993920; doi:10.1107/S2056989023001226)
Supplement: Supplementary file 6 [file e-79-00177-sup6.docx]

Supporting information for “Crystal structures of two 1,2,3,4tetrahydronaphthalenes obtained during efforts towards the total synthesis of elisabethin A: Synthetic and analytic details for compounds (2) –(8)

- 1. Methyl-(*R*)-3-((1*R*,4*S*)-6-methoxy-4,7-dimethyl-5,8-bis((triisopropylsilyl)oxy)-1,2,3,4-tetrahydronaphthalen-1-yl)butanoate **2**

A 50 mL Schlenk flask was equipped with known compound **1** ([doi.org/10.1021/acs.joc.2c01914](https://doi.org/10.1021/acs.joc.2c01914)) (111 mg, 0.175 mmol, 1 equiv.) in 15 mL dry ethyl acetate. The colorless solution was schlenked 10 times then Pd/C (21 mg, 19 µmol, 11 mol %) was added. The atmosphere was exchanged to H_2_ *via* vacuum/H_2_ backfill (5 times) and the mixture was heated to 50° C overnight. The next day another portion of Pd/C (37 mg, 35 µmol, 20 mol %) was added and the flask was purged with a fresh H_2_-ballon. Then the reaction was again heated overnight. This was repeated two more times and after 4 days NMR quench confirmed full conversion. The atmosphere was exchanged to Argon by vacuum/Argon backfill (5 times) and the black suspension was filtered over silica. Desired product was obtained as pale-yellow oil which solidified on standing in 89 % yield (99 mg, 0.156 mmol). A crystal of X-ray quality could be obtained by slow evaporation from DCM. ^1^H-NMR (400 MHz, CDCl_3_): δ= 3.60 (s, 3H), 3.59 (s, 3H), 3.21 – 3.12 (m, 1H), 2.99 – 2.91 (m, 1H), 2.43 – 2.30 (m, 1H), 2.23 – 2.11 (m, 4H), 2.03 (dd, *J* = 14.9, 11.2 Hz, 1H), 1.96 – 1.85 (m, 1H), 1.83 – 1.73 (m, 2H), 1.49 – 1.41 (m, 1H), 1.40 – 1.23 (m, 6H), 1.14 – 1.01 (m, 39H), 0.86 (d, *J* = 6.8 Hz, 3H); ^13^C-NMR (101 MHz, CDCl_3_): δ= 174.5, 148.0, 147.8, 141.5, 132.5, 125.2, 119.6, 60.4, 51.4, 39.6, 37.1, 34.1, 27.8, 25.8, 23.1, 18.6, 18.3, 18.2, 18.1, 18.0, 14.6, 14.1, 11.6.

[α]_D_^20^= +52.47 (c 1.0, CH_2_Cl_2_).

**Scheme 1**: Synthetic sequence toward compound **8**

- 1. (*R*)-1-((1*S*,4*S*)-6-methoxy-4,7-dimethyl-5,8-bis((triisopropylsilyl)oxy)-1,4-dihydronaphthalen-1-yl)ethane-1,2-diol **4**

25 mL round bottom flask was equipped with known compound **3** ([doi.org/10.1021/acs.joc.2c01914](https://doi.org/10.1021/acs.joc.2c01914)) (79 mg, 0.141 mmol, 1 equiv.) in 2.6 mL acetone and 0.13 mL H_2_O. NMO monohydrate (29 mg, 0.212 mmol, 1.5 equiv.) was added to the pale-yellow solution, followed by 2,6-lutidine (33µL, 0.283 mmol, 2 equiv.). Subsequently, OsO_4_ (2.5 % in *t*-BuOH, 37 µL, 2.8 µmol, 2 mol %) was added and the dark mixture was stirred. After 1.5 hours, TLC (petroleum ether/ ethyl acetate 50:1) confirmed full conversion. The reaction was quenched by addition of saturated Na_2_S_2_O_3_ solution, and the aqueous layer was extracted with Et_2_O four times. The combined organic layer was washed with Brine and dried over MgSO4. Solvents were removed in vacuo and the crude yellow oil was flashed over silica. Desired product was obtained in 92 % yield (77 mg, 0.130 mmol) as an inseparable 14:1 mixture of diastereomers. ^1^H-NMR (600 MHz, CD_2_Cl_2_): δ= 5.93 (ddd, *J* = 10.3, 4.1, 1.7 Hz, 1H), 5.87 (ddd, *J* = 10.3, 3.6, 1.5 Hz, 1H), 4.05 (ddd, *J* = 8.0, 4.9, 3.1 Hz, 1H), 3.94 – 3.90 (m, 1H), 3.62 (s, 3H), 3.55 – 3.48 (m, 1H), 3.27 (dd, *J* = 11.4, 7.9 Hz, 1H), 3.04 (dd, *J* = 11.4, 3.1 Hz, 1H), 2.61 (broad s, 1H), 2.19 (s, 3H), 1.71 (broad s, 1H), 1.39 – 1.31 (m, 6H), 1.21 (d, *J* = 6.7 Hz, 3H), 1.11 (dd, *J* = 9.6, 7.5 Hz, 18H), 1.08 (dd, *J* = 7.6, 4.5 Hz, 18H); ^13^C-NMR (151MHz, CD_2_Cl_2_): δ= 149.1, 147.4, 142.3, 132.2, 130.7, 121.8, 121.1, 121.0, 74.6, 64.3, 60.7, 40.3, 31.7, 24.5, 18.4, 18.3, 18.2, 18.1, 14.8, 14.3, 11.5; HRMS (ESI): exact mass calculated for C_33_H_61_O_5_Si_2_ [(M + H)^+^], 593.4058; found 593.4006; [α]_D_^20^= +142.69 (c 1.0, CH_2_Cl_2_).

- 1. (1*S*,4*S*)-6-methoxy-4,7-dimethyl-5,8-bis((triisopropylsilyl)oxy)-1,4-dihydronaphthalene-1-carbaldehyde **5**

A 10 mL round bottom flask was equipped with diol **4** (47 mg, 79 µmol, 1 equiv.) in 1.6 mL MeOH and 0.1 mL H_2_O was added. NaIO_4_ (51 mg, 0.238 mmol, 3 equiv.) was added to the clear solution causing precipitation after two minutes. The turbid mixture was stirred for 45 minutes at which point TLC (petroleum ether/ ethyl acetate 5:1) confirmed full conversion. The reaction was diluted with ethyl acetate and filtered into a separation funnel. The organic layer was washed with Brine, dried over MgSO_4_, and concentrated in vacuo. Desired product was obtained in 85 % (38 mg, 68 µmol). ^1^H-NMR (600 MHz, CDCl_3_): δ= 9.07 (d, *J* = 2.7 Hz, 1H), 6.04 (ddd, *J* = 10.1, 4.3, 2.5 Hz, 1H), 5.59 (ddd, *J* = 10.0, 3.9, 1.4 Hz, 1H), 4.24 (ddd, *J* = 6.2, 3.6, 2.6 Hz, 1H), 3.61 – 3.55 (m, 1H), 2.20 (s, 3H), 1.37 – 1.28 (m, 6H), 1.24 (d, *J* = 6.8 Hz, 3H), 1.09 (dd, *J* = 7.5, 2.9 Hz, 18H), 1.07 – 1.02 (m, 18H); ^13^C-NMR (151MHz, CDCl_3_): δ= 200.3, 149.6, 147.8, 141.9, 134.5, 130.3, 120.7, 118.6, 117.2, 60.6, 50.1, 30.7, 24.2, 18.3, 18.2, 18.1, 18.1, 14.5, 14.1, 11.4; [α]_D_^20^= +15.66 (c 0.65, CH_2_Cl_2_).

- 1. (1*S*,4*S*)-6-methoxy-4,7-dimethyl-5,8-bis((triisopropylsilyl)oxy)-1,2,3,4-tetrahydronaphthalene-1-carbaldehyde **6**

A 10 mL Schlenk flask was equipped with aldehyde **5** (38 mg, 68 µmol, 1 equiv.) in 5 mL dry degassed DCM and Crabtree's catalyst (8 mg, 10 µmol, 15 mol %) was added. The flask was evacuated and backfilled with H_2_ eight times causing a color change from orange to yellow. The H_2_ valve was left open, and the reaction was stirred at room temperature. After 5 hours GC-MS confirmed full conversion (same R_f_ on TLC). The yellow solution was filtered through silica and solvents were evaporated. Desired product was obtained as orange oily solids in quantitative yield (38 mg, 68 µmol). ^1^H-NMR (600 MHz, CDCl_3_): δ= 9.56 (d, *J* = 1.2 Hz, 1H), 3.73 (dd, *J* = 6.3, 1.7 Hz, 1H), 3.64 (s, 3H), 3.15 (tp, *J* = 7.0, 3.4 Hz, 1H), 2.29 (dtd, *J* = 13.5, 3.3, 1.5 Hz, 1H), 2.20 (s, 3H), 1.90 – 1.81 (m, 1H), 1.55 (dq, *J* = 8.6, 3.0 Hz, 2H), 1.35 – 1.24 (m, 6H), 1.15 (d, *J* = 6.9 Hz, 3H), 1.11 – 1.01 (m, 36H); ^13^C-NMR (151MHz, CDCl_3_): δ= 203.8, 149.0, 147.8, 142.0, 133.4, 128.0, 123.6, 120.6, 120.0, 118.1, 60.6, 47.0, 27.6, 26.4, 21.7, 18.3, 18.2, 18.0, 18.0, 17.4, 14.5, 14.1, 11.5.

- 1. Methyl-(*E*)-3-((1*R*,4*S*)-6-methoxy-4,7-dimethyl-5,8-bis((triisopropylsilyl)oxy)-1,2,3,4-tetrahydronaphthalen-1-yl) acrylate **7**

A 25 mL Schlenk with NaH (55 %, 46 mg, 1,06 mmol, 2 equiv.) in 3 mL dry THF and the grey suspension was cooled to 0° C. After 2 minutes, trimethyl phosphonoacetate was added dropwise causing slight gas formation. After full addition, the white slurry reaction mixture was removed from cooling and stirred at room temperature for 25 minutes. Then aldehyde **6** (298 mg, 0.53 mmol, 1 equiv.) dissolved in 3 ml dry THF was added dropwise. After 2 hours, TLC (petroleum ether/ ethyl acetate 20:1) did not show any conversion. To dissolve the slurry 1.2 mL freshly distilled DMPU was added, and the reaction mixture was stirred for another 16 hours. After TLC (petroleum ether/ ethyl acetate 20:1) confirmed full conversion the red-brown reaction was quenched by addition of saturated NH_4_Cl solution, and the aqueous layer was extracted with ethyl acetate three times. The combined organic layer was washed with H_2_O and dried over MgSO_4_. The crude residue was purified by flash chromatography (petroleum ether: ethyl acetate 20:1) and pure product was obtained as yellow oil in 87 % yield (286 mg, 0.46 mmol). ^1^H-NMR (600 MHz, CDCl_3_): δ= 7.00 (dd, *J* = 15.7, 4.7 Hz, 1H), 5.13 (dd, *J* = 15.7, 1.8 Hz, 1H), 3.86 – 3.81 (m, 1H), 3.65 (s, 3H), 3.62 (s, 3H), 3.15 – 3.08 (m, 1H), 2.15 (s, 3H), 2.06 – 1.97 (m, 1H), 1.76 – 1.69 (m, 2H), 1.48 – 1.42 (m, 1H), 1.37 – 1.23 (m, 6H), 1.14 (d, *J* = 7.0 Hz, 3H), 1.10 – 1.02 (m, 36H); ^13^C-NMR (151 MHz, CDCl_3_): δ= 167.7, 153.8, 148.7, 147.5, 141.7, 132.4, 121.9, 121.1, 119.7, 60.5, 51.4, 36.1, 27.9, 24.9, 22.2, 21.8, 18.3, 18.2, 18.1, 14.5, 14.1, 11.4.

- 1. Methyl (*E*)-3-((1*R*,4*S*)-8-hydroxy-6-methoxy -4,7-dimethyl-5-((triisopropylsilyl)oxy)-1,2,3,4- tetrahydro naphthalen-1-yl)acrylate **8**

A 25 mL round bottom flask was equipped with ester **7** (143 mg, 0.231 mol, 1 equiv.) and acetic acid (66 µL, 1.16 mmol, 5 equiv.) in 0.5 mL dry THF was added. After 5 minutes TBAF (1.0 M in THF, 289 µl, 289 µmol, 1.25 equiv.) was added dropwise. The yellow solution was stirred at room temperature for 6 hours until TLC (petroleum ether: ethyl acetate 10:1) confirmed full conversion. The reaction was quenched with saturated NaHCO_3_ solution and the aqueous layer was extracted with Et_2_O three times. The combined organic layer was dried over MgSO_4_ and concentrated in vacuo. The crude material was purified by column chromatography (3.4 g silica, petroleum ether: ethyl acetate 20:1) and desired product was collected as orange oil which solidified upon standing in 60 % yield (64 mg, 0.138 mmol).

A crystal of X-ray quality could be obtained by slow evaporation from DCM. ^1^H-NMR (400 MHz, CDCl_3_): δ= 7.07 (dd, *J* = 15.6, 6.1 Hz, 1H), 5.44 (dd, *J* = 15.6, 1.6 Hz, 1H), 3.78 – 3.73 (m, 1H), 3.69 (s, 3H), 3.66 (s, 3H), 3.20 – 3.09 (m, 1H), 2.16 – 2.05 (m, 4H), 1.82 – 1.69 (m, 2H), 1.50 (ddd, *J* = 15.2, 5.5, 3.3 Hz, 1H), 1.39 – 1.29 (m, 3H), 1.19 (d, *J* = 6.9 Hz, 3H), 1.08 (dd, *J* = 8.8, 7.5 Hz, 18H); ^13^C-NMR (101 MHz, CDCl_3_): δ= 167.4, 152.3, 148.7, 146.0, 141.4, 132.8, 121.3, 117.9, 115.5, 60.9, 51.6, 35.5, 27.8, 24.7, 22.2, 21.5, 18.3, 18.2, 14.0, 9.2.
